# Survival outcomes after breast-conserving surgery plus radiotherapy compared with mastectomy in breast ductal carcinoma in situ with microinvasion

**DOI:** 10.1038/s41598-022-24630-7

**Published:** 2022-11-22

**Authors:** Lin-Yu Xia, Wei-Yun Xu, Qing-Lin Hu

**Affiliations:** 1 Department of Thyroid and Breast Surgery, The First Affiliated Hospital of Chengdu Medical College, 278 Baoguang Avenue Middle Section, Xindu District, Chengdu, 610500 Sichuan China; 2 Department of Breast Surgery, Mianyang Central Hospital, Mianyang, Sichuan China

**Keywords:** Cancer, Medical research, Oncology

## Abstract

Ductal carcinoma in situ with microinvasion (DCIS-MI) is a subtype of breast cancer with a good prognosis, for which both breast conserving surgery plus radiotherapy (BCS + RT) and mastectomy are feasible surgical methods, but no clear conclusion has been made on the choice of these treatments. We used the Surveillance, Epidemiology and End Results database to extract 5432 DCIS-MI patients. Participants were divided into the BCS + RT group and the mastectomy group. We compared the overall survival (OS) and breast cancer-specific survival (BCSS) of the two groups using the Kaplan–Meier method and Cox regressions before and after propensity score matching (PSM). Before PSM, both univariate and multivariate analyses showed that BCS + RT group had significantly higher OS and BCSS compared with patients in the mastectomy group (*P* < 0.001). After PSM, the multivariate analysis showed that compared with mastectomy, the BCS + RT showed significantly higher OS and BCSS (HR = 0.676, 95% CI = 0.540–0.847, *P* < 0.001; HR = 0.565,95% CI = 0.354–0.903, *P* = 0.017). In addition, the subgroup analysis showed that BCS + RT is at least equivalent to mastectomy with respect to OS and BCSS in any subgroup. For patients with DCIS-MI, the prognosis of BCS + RT was superior to mastectomy.

## Introduction

Ductal carcinoma in situ with microinvasion (DCIS-MI) is a special type of breast cancer, accounting for 0.6–3.4% of breast cancer^1,2^. It refers to cancer cells breaking through the basement membrane to infiltrate adjacent tissues, but the maximum lesion scope is less than 1 mm^3,4^. According to the American Joint Committee on Cancer (AJCC), lesions that meet this definition are regarded as a subtype of stage T1 breast cancer and classified as T1mic stage^5^. Most scholars believe that DCIS-MI is the intermediate stage of DCIS and invasive ductal carcinoma (IDC), with a prognosis between the two^6–9^. However, some scholars believe that DCIS-MI has the same prognosis as DCIS^10,11^. The early stage proposed by DCIS-MI lacks a unified diagnostic standard, and the study sample size is small. Therefore, there are many controversies in the treatment.

Currently, many studies have demonstrated that for early breast cancer, patients receiving BCS + RT have the same prognosis as patients receiving mastectomy^12–14^. Mamtani et al. compared the prognosis of BCS + RT and mastectomy in patients with DCIS and found that BCS + RT was superior to mastectomy in OS or DFS of DCIS^15^. Both BCS + RT and mastectomy are currently available surgical methods for DCIS-MI. However, considering the good prognosis of DCIS-MI and patients’ postoperative life quality, it is worth exploring whether BCS + RT is the best choice for DCIS-MI. At present, there are few studies on the surgical methods of DCIS-MI^16–18^. The prognosis of BCS + RT or mastectomy for patients with DCIS-MI is still unclear. We conducted this study to determine which surgical procedure is better for patients with DCIS-MI. This study compared the long-term outcomes of patients with DCIS-MI receiving BCS + RT and mastectomy using the SEER database.

## Materials and methods

### Patients

This study was conducted using the SEER database published in November 2018. Patients who were diagnosed with DCIS-MI from 2000 to 2014 were eligible for recruitment. The inclusion criteria included: (1) 20–79 years old; (2) female; (3) a mastectomy or breast-conserving surgery was performed. Exclusion criteria included: (1) patients with tumor metastasis; (2) patients combined with other malignant tumors; (3) patients who did not receive radiotherapy after breast-conserving surgery.

### Data collection and outcome measures

We collected the following factors: year of diagnosis, age, race, marital status, histological grade (well differentiated, moderately differentiated, poorly differentiated, undifferentiated), lymph node status, estrogen receptor (ER), progesterone receptor (PR), surgical method, chemotherapy, and radiotherapy. Our study’s main outcomes were OS and BCSS, OS was defined as the time from the date of diagnosis to the date of death, and BCSS was measured from the date of diagnosis to the date of death due to breast cancer.

### Statistical analysis

Propensity score matching (PSM) was applied to create a matched pair between the two groups to eliminate the selection bias of this study population^19^. We performed PSM for all the variables included in the study. Landmark analysis was used to eliminate a lead time bias among the propensity-matched cohort^20^. With the landmark, analysis was restricted to patients who survived to 6 months without death. X^2^ test was used to compare the distribution of the clinical and pathological features between the two groups before and after PSM. The OS and BCSS survival curves were plotted through the Kaplan–Meier method and compared by the log-rank test. The cox regression model was used for the univariate and multivariate analyses of the BCSS and OS. All *P* values were two-sided, and *P* < 0.05 was considered significant. The SPSS 20.0 (IBM SPSS Statistics, Chicago, IL, US) was used for these analyses.

### Ethics approval and consent to participate

All patients were collected from the SEER database, and all of them have given prior informed consent to being registered in it. The study was approved by the Ethics Committee of The First Affiliated Hospital of Chengdu Medical College and was complied with the Declaration of Helsinki.

## Results

### Baseline characteristics

In total, 5432 patients with DCIS-MI from 2000 to 2014 were included in the study through the SEER database. We divided the patients into two groups: BCS + RT group (2834,52.17%) and mastectomy group (2598,47.83%). Table 1 summarizes the patient clinical characteristics of the two groups. Compared with mastectomy group, the patients in the BCS group were older (78.9% vs. 64.2%; *P* < 0.001) and had a lower histological grade (grade I + II, 65.6% vs. 56.4%; *P* < 0.001), less lymph node metastasis (N0, 96.7% vs. 87.5%; *P* < 0.001). Further, the BCS group had a higher ER (76.7% vs. 67.4%; *P* < 0.001) and PR (62.9% vs. 55.4%; *P* < 0.001) positive rates and were less likely to receive chemotherapy (6.2% vs. 14.8%; *P* < 0.001). After PSM, the two groups consisted of 1902 pairs. There was no significant difference in clinicopathological characteristics between the two groups.

**Table 1:** Baseline characteristics of the study population and tumor

Characteristics		Before PSM		*P*	After PSM		*P*
		BCS + RT (n,%)	Mastectomy (n,%)		BCS + RT (n,%)	Mastectomy (n,%)	
No. of patients		2834	2598(47.83%)		1902	1902	
Year of diagnosis	2004–2009	1304(46%)	1181(45.5%)	0.682	851(44.7%)	849(44.6%)	0.948
	2010–2014	1530(54%)	1417(54.5%)		1051(55.3%)	1053(55.4%)	
Age (years)	20–49	597(21.1%)	931(35.8%)	** < 0.001**	488(25.7%)	489(25.7%)	0.970
	50–80	2237(78.9%)	1667(64.2%)		1414(74.3%)	1413(74.3%)	
Race	White	2179(76.9%)	1944(74.8%)	0.205	1476(77.6%)	1481(77.9%)	0.888
	Black	333(11.8%)	335(12.9%)		210(11.0%)	214(11.2%)	
	Other	322(11.4%)	319(12.3%)		216(11.4%)	207(10.9%	
Marital status	Married	963(34%)	840(32.3%)	0.198	628(33.0%)	622(32.7%)	0.836
	Not married	1871(66%)	1758(67.7%)		1274(67.0%)	1280(67.3%)	
Grade	I	748(26.4%)	473(18.2%)	** < 0.001**	408(21.5%)	408(21.5%)	1
	II	1112(39.2%)	993(38.2%)		761(40.0%)	761(40.0%)	
	III	848(29.9%)	995(38.3%)		652(34.3%)	652(34.3%)	
	IV	126(4.4%)	137(5.3%)		81(4.3%)	81(4.3%)	
Nodal status	N0	2741(96.7%)	2274(87.5%)	** < 0.001**	1840(96.7%)	1841(96.8%)	1
	N1	78(2.8%)	273(10.5%)		55(2.9%)	54(2.8%)	
	N2	11(0.4%)	35(1.3%)		6(0.3%)	6(0.3%)	
	N3	4(0.1%)	16(0.6%)		1(0.1%)	1(0.1%)	
ER	Negative	660(23.3%)	846(32.6%)	** < 0.001**	523(27.5%)	523(27.5%)	1
	Positive	2174(76.7%)	1752(67.4%)		1379(72.5%)	1379(72.5%)	
PR	Negative	1050(37.1%)	1159(44.6%)	** < 0.001**	752(39.5%)	756(39.7%0	0.895
	Positive	1784(62.9%)	1439(55.4%)		1150(60.5%)	1146(60.3%)	
Chemotherapy	yes	177(6.2%)	384(14.8%)	** < 0.001**	102(5.4%)	103(5.4%)	0.943
	no	2657(93.8%)	2214(85.2%)		1800(94.6%)	1799(94.6%)	
Radiotherapy	yes	2834(100%)	184(7.1%)		1902(100%)	85(4.5%)	
	no	0(0%)	2414(92.9%)		0(0%)	1817(95.5%)	

*PSM*  propensity score matching; *BCS + RT*  Breast conserving surgery plus radiotherapy. Significant values are in [bold].

### Prognostic factors associated with OS and BCSS

**Figure 1:**
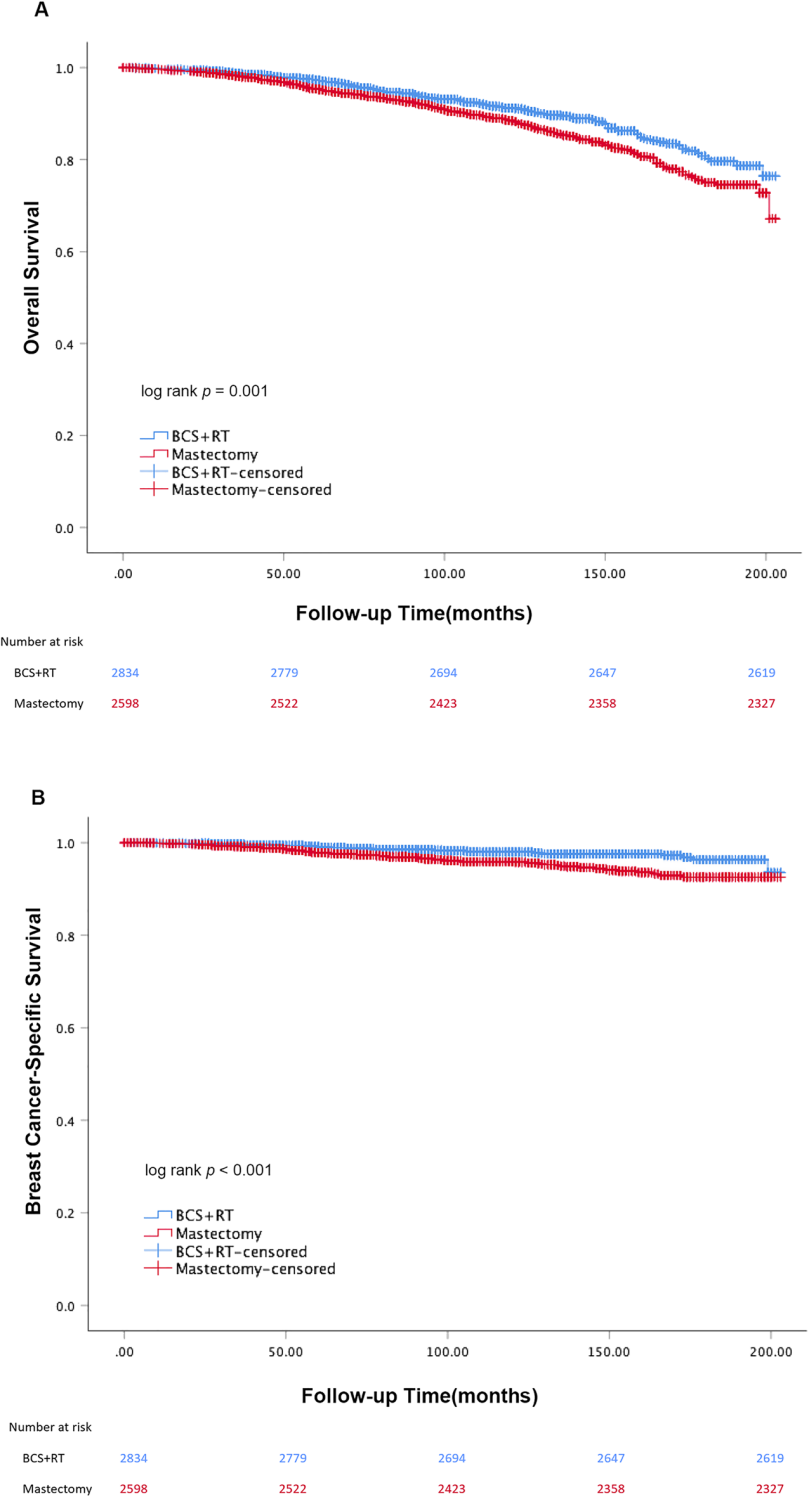
Kaplan–Meier curves of OS (**A**) and BCSS (**B**) for unmatched cohorts.

Before PSM, the median follow-up time for these patients was 101 months. The 5-year and 10-year OS for patients in BCS + RT and mastectomy groups were 97.3% vs. 95.4% and 91.2% vs. 88.5% respectively (log-rank *P* = 0.001, Fig. 1A). The 5-year and 10-year BCSS for patients in BCS + RT and mastectomy groups were 99.1% vs. 97.8% and 98.0% vs. 95.9% (log-rank *P* < 0.001, Fig. 1B). After adjusting for the prognostic variables in the univariate analysis (Supplementary Table 1), the multivariate analysis indicated that black race and patients with more lymph node metastases are associated with poor OS and BCSS (all *P* < 0.05). Besides, patients at a younger age and not married had better OS relatively while patients without chemotherapy had lower BCSS (all *p* < 0.05). The BCS + RT group showed significantly higher OS and BCSS compared with patients in the mastectomy group (HR = 0.686, 95% CI = 0.571–0.825, *P* < 0.001; HR = 0.596, 95% CI = 0.411–0.865, *P* = 0.007) (Table 2).

**Table 2:** Prognostic factors for OS and BCSS in multivariate analysis

Characteristics		OS	*P*	BCSS	*P*
		HR(95%CI)		HR(95%CI)	
Year of diagnosis	2000–2007	Ref	Ref	Ref	Ref
	2008–2014	0.795(0.625–1.011)	0.061	0.902(0.603–1.349)	0.614
Age (years)	20–49	Ref	Ref	Ref	Ref
	50–80	2.806(2.158–3.648)	** < 0.001**	0.875(0.611–1.253)	0.465
Race	White	Ref	Ref	Ref	Ref
	Black	1.522(1.199–1.933)	** < 0.001**	1.900(1.261–2.863)	**0.002**
	Other	0.696(0.479–1.012)	0.058	0.782(0.393–1.555)	0.483
Marital status	Married	Ref	Ref	Ref	Ref
	Not married	0.615(0.513–0.738)	** < 0.001**	0.730(0.514–1.037)	0.079
Grade	I	Ref	Ref	Ref	Ref
	II	0.859(0.677–1.090)	0.212	0.904(0.543–1.504)	0.697
	III	0.808(0.623–1.048)	0.108	1.191(0.716–1.980)	0.500
	IV	0.735(0.479–1.128)	0.159	0.987(0.446–2.187)	0.975
Nodal status	N0	Ref	Ref	Ref	Ref
	N1	1.204(0.829–1.747)	0.330	1.941(1.113–3.384)	**0.019**
	N2	2.248(1.079–4.684)	**0.031**	2.961(1.163–7.540)	**0.023**
	N3	5.600(2.687–11.672)	** < 0.001**	10.648(4.298–26.381)	** < 0.001**
ER	Positive	Ref	Ref	Ref	Ref
	Negative	0.998(0.760–1.312)	0.990	1.354(0.838–2.189)	0.216
PR	Positive	Ref	Ref	Ref	Ref
	Negative	0.868(0.675–1.115)	0.268	0.692(0.444–1.078)	0.103
Chemotherapy	yes	Ref	Ref	Ref	Ref
	no	0.980(0.690–1.393)	0.910	1.747(1.042–2.930)	**0.034**
Surgical method	BCS + RT	0.686(0.571–0.825)	** < 0.001**	0.596(0.411–0.865)	**0.007**
	Mastectomy	Ref	Ref	Ref	Ref

*OS*  overall survival; *BCSS*  breast cancer-specific survival. Significant values are in [bold].

**Figure 2:**
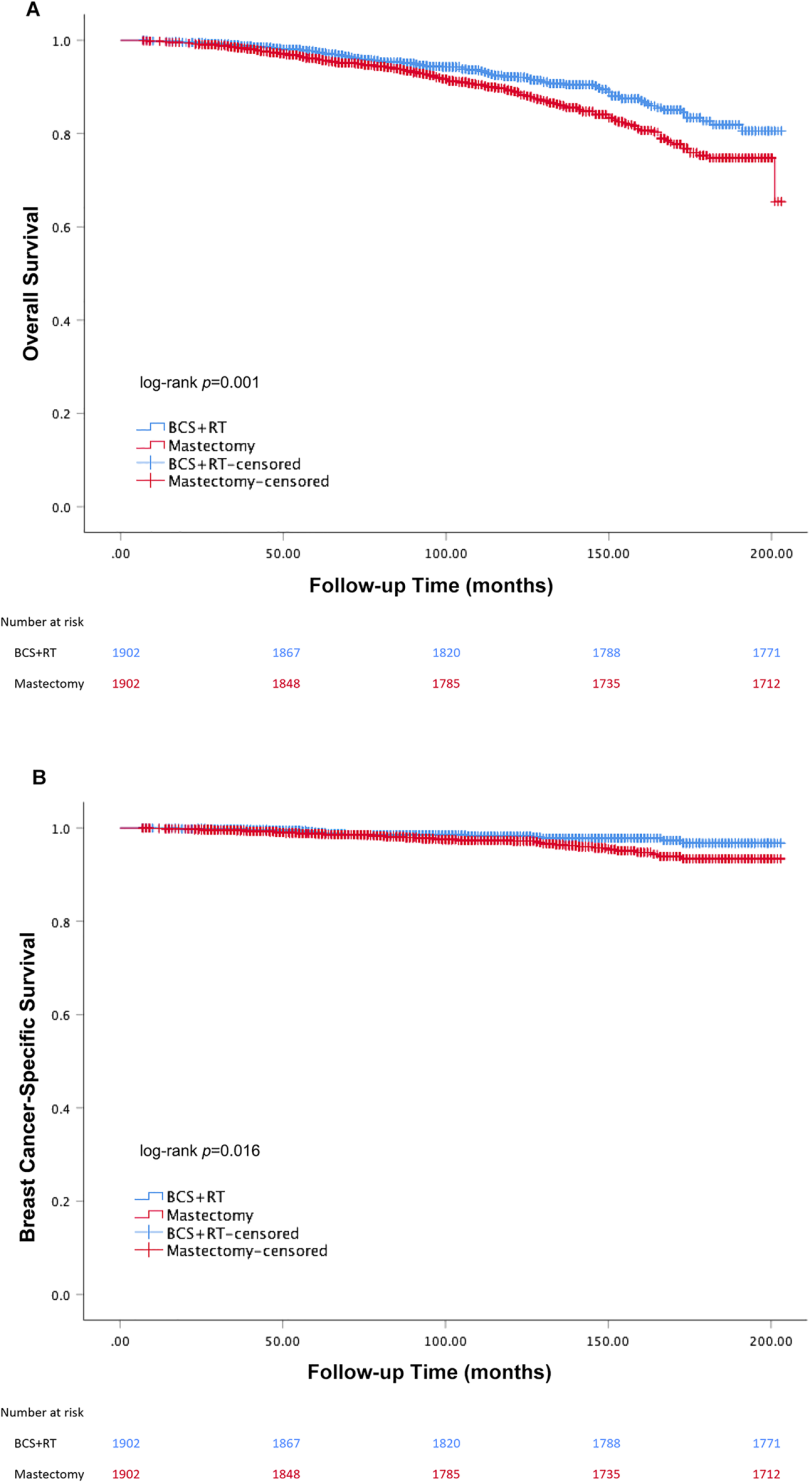
Kaplan–Meier curves of OS (**A**) and BCSS (**B**): propensity matched landmark analysis.

After PSM with a 6-month landmark, the 5-year and 10-year OS for patients in BCS + RT and mastectomy groups were 97.4% vs. 95.9% and 92.1% vs. 89.1% respectively (log-rank *P* = 0.001, Fig. 2A). The 5-year and 10-year BCSS for patients in BCS + RT and mastectomy groups were 99.1% vs. 98.7% and 98.2% vs. 97.4% (log-rank *P* = 0.016, Fig. 2B). Adjusting for the significant prognostic variables in univariate analysis (Supplementary Table 2), the multivariate cox regression analysis showed that the BCS + RT group showed significantly higher OS and BCSS compared with mastectomy group (HR = 0.676, 95% CI = 0.540–0.847, *P* < 0.001; HR = 0.565,95% CI = 0.354–0.903, *P* = 0.017). Patients at a younger age and not married had better OS while black race and patients with more lymph node metastases had poor OS and BCSS (all *P* < 0 0.05). Besides, grade III demonstrated a worse effect on BCSS (HR = 2.210, 95% CI = 1.022–4.778, *P* = 0.044) (Table 3).

**Table 3:** Prognostic factors for OS and BCSS in multivariate analysis after PSM.

Characteristics		OS	*P*	BCSS	*P*
		HR(95%CI)		HR(95%CI)	
Year of diagnosis	2000–2007	Ref	Ref	Ref	Ref
	2008–2014	0.751(0.544–1.018)	0.065	0.902(0.517–1.573)	0.717
Age (years)	20–49	Ref	Ref	Ref	Ref
	50–80	3.763(2.539–5.578)	** < 0.001**	1.007(0.592–1.711)	0.980
Race	White	Ref	Ref	Ref	Ref
	Black	1.634(1.200–2.226)	**0.002**	2.203(1.240–3.915)	**0.007**
	Other	0.534(0.311–0.916)	0.053	0.540(0.168–1.737)	0.301
Marital status	Married	Ref	Ref	Ref	Ref
	Not married	0.601(0.478–0.755)	** < 0.001**	0.703(0.432–1.146)	0.158
Grade	I	Ref	Ref	Ref	Ref
	II	0.900(0.666–1.215)	0.490	1.630(0.759–3.502)	0.210
	III	0.868(0.626–1.202)	0.394	2.210(1.022–4.778)	**0.044**
	IV	0.738(0.413–1.318)	0.304	1.383(0.372–5.142)	0.629
Nodal status	N0	Ref	Ref	Ref	Ref
	N1	1(0.509–1.964)	1	4.001(1.607–9.961)	**0.003**
	N2	7.004(1.931–25.403)	**0.003**	12.960(2.953–56.873)	**0.001**
	N3	36.754(4.349–310.608)	**0.001**	53.355(4.923–578.230)	**0.001**
ER	Positive	Ref	Ref	Ref	Ref
	Negative	0.897(0.622–1.292)	0.558	1.418(0.709–2.835)	0.323
PR	Positive	Ref	Ref	Ref	Ref
	Negative	0.993(0.706–1.396)	0.966	0.710(0.375–1.342)	0.292
Chemotherapy	yes	Ref	Ref	Ref	Ref
	no	0.916(0.505–1.661)	0.773	1.241(0.503–3.065)	0.639
Surgical method	BCS + RT	0.676(0.540–0.847)	**0.001**	0.565(0.354–0.903)	**0.017**
	Mastectomy	Ref	Ref	Ref	Ref

*OS*  overall survival; *BCSS*  breast cancer-specific survival; *PSM*  propensity score matching. Significant values are in [bold].

### Subgroup analysis of OS and BCSS

**Figure 3:**
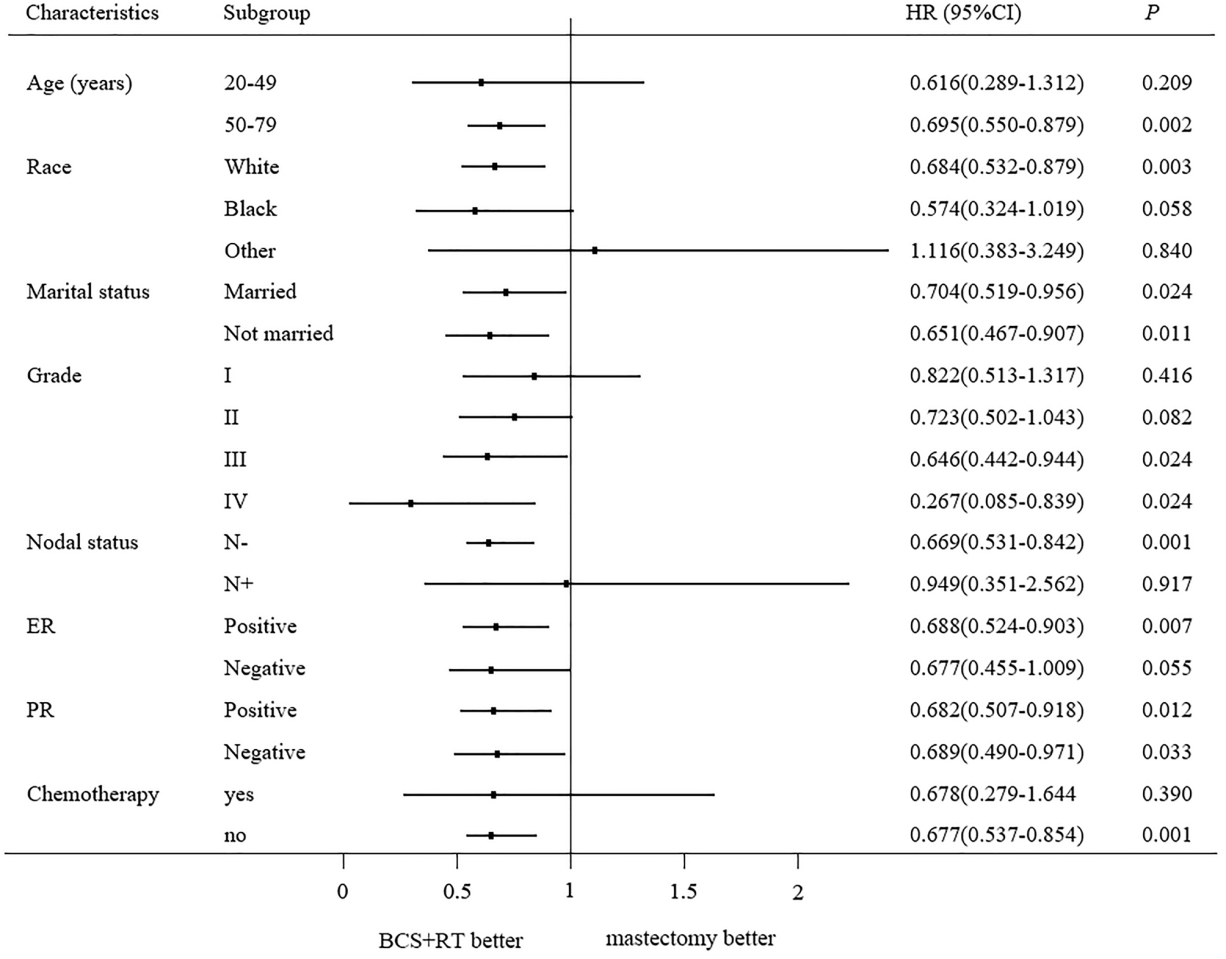
The forest plot of HR for OS between the BCS + RT group and mastectomy group according to different characteristics.

**Figure 4:**
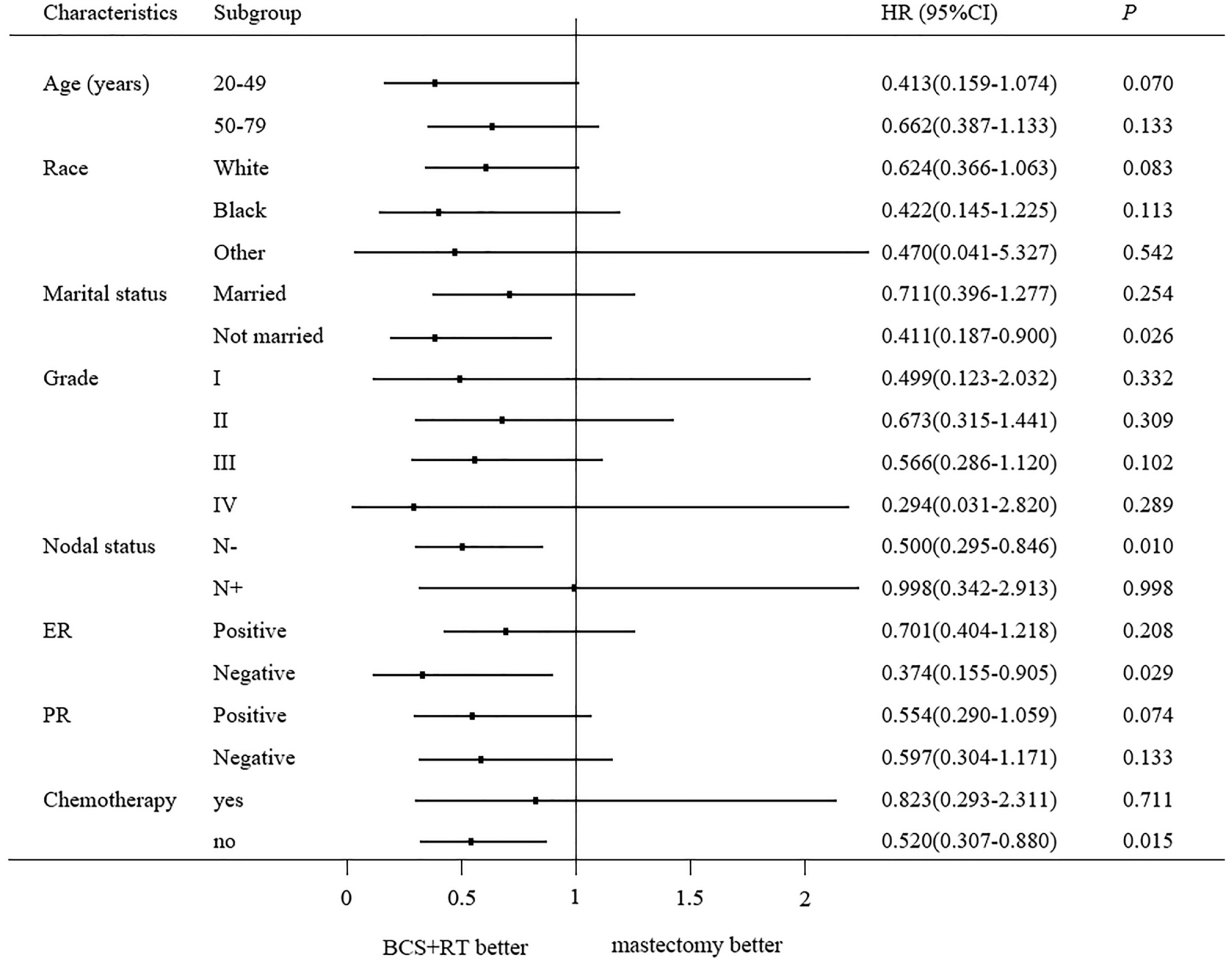
The forest plot of HR for BCSS between the BCS + RT group and mastectomy group according to different characteristics.

To further explore possible factors affecting the overall survival time for patients who had undergone two types of surgery, we performed a subgroup analysis of all patients after PSM. BCS + RT group showed significantly higher OS than the mastectomy group for patients aged between 50–79 years, patients married or unmarried, the white race group, patients with grade III + IV, patients with lymph nodal negative, patients with ER positive, patients with PR- positive or negative and those who did not receive chemotherapy (all *P* < 0.05). There was no difference significantly observed in OS in other subgroups (Fig. 3). The BCS + RT group also showed BCSS benefits in patients who were not married, patients with lymph nodal negative, patients with ER- negative, and those who did not receive chemotherapy (Fig. 4). Further, the OS and BCSS outcomes of mastectomy were not better than BCS + RT in any subgroup.

## Discussion

DCIS-MI is a special type of breast cancer, and there is little evidence on the prognosis of patients with DCIS-MI undergoing BCS + RT and mastectomy. We found that the prognosis of patients with DCIS-MI after mastectomy is not better than those of BCS + RT in any subgroup by using the SEER database.

In the NCCN guidelines, DCIS-MI is classified as early-stage invasive breast cancer. All surgical options for early-stage invasive breast cancer are unified. There is no special explanation for the surgical options for DCIS-MI. In our study, 71.9% of patients with DCIS-MI were older than 50 years. Besides, patients with DCIS-MI had few lymph node metastases (7.7%), low histological grade (61.3% in GI + II), and high positive rates of ER and PR (72.3% and 59.3%), which was consistent with other studies^21,22^. These results indicate that most of the DCIS-MI have a good prognosis.

Although the clinicopathological features of DCIS-MI indicate a good prognosis, at present, there are still a large number of DCIS-MI patients undergoing a mastectomy. In our study, 47.83% of the patients received mastectomy. In Eastern countries, the proportion of patients with DCIS-MI undergoing mastectomy is higher, even as high as 80%^23,24^. At present, there are few studies on the prognosis of DCIS-MI after surgery. Mamtani et al. investigated the prognosis of DCIS ± MI after mastectomy. It proved that distant disease-free survival after mastectomy for DCIS ± microinvasion is excellent among all age groups, and overall rates of locoregional recurrence after mastectomy for DCIS with or without microinvasion are low. Even in the age group with the highest recurrence rate, 10-year locoregional recurrence remains low at 4.2%^25^. Park et al. conducted a study on 3648 patients with DCIS younger than 40 years old, and the results showed that mastectomy does not offer survival benefits over BCS + RT^26^. Mamtani et al. also confirmed this^15^. The Yale School of Medicine retrospective clinical study included 72 patients with DCIS-MI and 321 patients with DCIS, all of whom received BCS + RT. There was no difference in regional recurrence rates after 10 years between the DCIS-MI group and the DCIS group (8.3% vs. 6.8%)^18^. DCIS-MI often has multiple minimally invasive foci, associated with a higher risk of ipsilateral recurrence^27,28^. The study by Si et al. showed that 35.1% of DCIS-MI Patients have multiple foci, which had a worse disease-free survival rate compared with one-focus patients (98.29 vs. 93.01%, *P* = 0.032)^24^. The safety of BCS for DCIS-MI with multiple minimally invasive foci is worth exploring. Rakovitch compared the local recurrence rate after BCS in DCIS-MI patients with one-focus and multiple foci^17^. The results showed that multiple foci of MI are associated with an increased risk of invasive local recurrence in women with DCIS treated with BCS, but treatment with the whole breast and boost RT can mitigate this risk. At present, there is no study comparing the prognosis of BCS + RT and mastectomy in DCIS-MI patients with multiple foci.

There are few studies comparing the prognosis of patients with DCIS-MI after BCS + RT and mastectomy. Bartova et al. compare the prognostic difference between BCS and mastectomy in DCIS-MI. They followed up on 41 patients with DCIS or DCIS-MI after BCS and mastectomy, and finally, only 27 patients completed the follow-up. There is no local recurrence occurred^16^. However, the sample size of this study was small, and no survival rate was reported. In our study, we observed that 95.5% of patients received mastectomy without RT. Thus we think that BCS + RT showed a better prognosis than mastectomy may due to RT. Studies have confirmed that RT can reduce the local recurrence of breast cancer after BCS. Fisher et al.^12^ showed that adjuvant radiotherapy after BCS could reduce the risk of recurrence by approximately 50%. The EBCTCG study also demonstrated this^29^. Rakovitch et al. proved that postoperative radiotherapy could reduce the local recurrence rate in patients with DCIS-MI^17^. Li et al. compared the difference in survival between DCIS-MI patients treated with BCS + RT (n = 74) and mastectomy without RT (n = 221). No survival difference was observed between the two groups^30^. In their study, none of the patients in the mastectomy group received radiotherapy, and the sample size of this study was small. We believe that further studies are needed to investigate the prognosis of DCIS-MI after different surgical methods.

Similar to the result of another study, chemotherapy cannot improve the survival of DCIS-MI in our study. Pu et al. proved that postoperative chemotherapy did not improve DFS in patients with DCIS-MI after mastectomy (HR = 1.50, 95% CI 0.29–7.87, *P* = 0.63)^31^. Chen et al. analyzed 3198 DCIS-MI patients and concluded that chemotherapy was an independent factor for worse BCSS (*P* = 0.008), and there was no statistical significance for OS (*P* = 0.248) in patients with DCIS-MI^32^. However, further studies are needed to verify whether chemotherapy is beneficial to patients with DCIS-MI.

Our study had several limitations. Firstly, the SEER database did not provide detailed information on breast multiple lesions and lacks data on the size of the DCIS in DCIS-MI and postoperative local recurrence. Secondly, there is no information on endocrine therapy and targeted therapy in the SEER database. Despite these limitations, the sample size of our study was large and the follow-up time was long. In the research method, we also used the propensity-matched landmark analysis to minimize the confounding factors. All these guarantee the reliability of our research results. We not only analyzed the OS and BCSS of the two groups but also performed subgroup analysis. We found that in any subgroup, the OS and BCSS results of mastectomy were not better than BCS + RT. There is little evidence on the prognosis of patients with DCIS-MI undergoing BCS + RT and mastectomy at present. The sample sizes of the studies were all small, and one of the studies did not report the survival rate. Therefore, our research is still very valuable and can provide a theoretical basis for the selection of surgical methods for DCIS-MI.

## Conclusion

This population-based study revealed that the prognosis of patients who were diagnosed with DCIS-MI receiving mastectomy was not better than those receiving BCS + RT. We think that BCS + RT should be considered preferentially in DCIS-MI. However. BCS + RT is appropriate in patients with a limited extent of disease. The surgical method should be selected carefully when the tumor has multiple foci or with a large mass.

## Supplementary Information

Supplementary Information.

## Data Availability

The datasets generated and/or analysed during the current study are available in the [Surveillance, Epidemiology, and End Results (SEER)] repository, [https://seer.cancer.gov/data/]”.SEER*Stat Database: Incidence‐SEER 18 Regs Custom Data (with additional treatment fields).
