# Supplementary material for: Survival outcomes after breast-conserving surgery plus radiotherapy compared with mastectomy in breast ductal carcinoma in situ with microinvasion
Source: Sci Rep. 2022 Nov 22;12:20132. doi: 10.1038/s41598-022-24630-7 (PMC9684534; doi:10.1038/s41598-022-24630-7)
Supplement: Supplementary file 1 — Supplementary Information. [file 41598_2022_24630_MOESM1_ESM.pdf]

Survival outcomes after Breast-conserving surgery plus radiotherapy compared with mastectomy in Breast ductal carcinoma in situ with microinvasion

Lin-Yu Xia[1]* , Wei-Yun Xu[2] , Qing-Lin Hu[1]

Supplementary Table 1 Prognostic factors for OS and BCSS in univariate analysis

Characteristics		OS		BCSS	
		HR(95%CI)	*P*	HR(95%CI)	*P*
Year of diagnosis	2000-2007	Ref.	Ref.	Ref.	Ref.
	2008-2014	0.836(0.658-1.061)	0.141	0.865(0.579-1.291)	0.477
Age (years)	20-49	Ref.	Ref.	Ref.	Ref.
	50-80	2.709(2.093-3.507)	**<0.001**	0.724(0.513-1.023)	0.067
Race	White	Ref.	Ref.	Ref.	Ref.
	Black	1.683(1.336-2.120)	**<0.001**	2.456(1.666-3.619)	**<0.001**
	Other	0.620(0.427-0.899)	**0.012**	0.691(0.349-1.370)	0.29
Marital status	Married	Ref.	Ref.	Ref.	Ref.
	Not married	0.561(0.470-0.671)	**<0.001**	0.689(0.491-0.966)	**0.031**
Grade	I	Ref.	Ref.	Ref.	Ref.
	II	0.849(0.672-1.074)	0.172	1.003(0.607-1.656)	0.992
	III	0.849(0.670-1.075)	0.173	1.619(1.012-2.588)	**0.044**
	IV	0.753(0.498-1.138)	0.178	1.255(0.583-2.703)	0.561
Nodal status	N0	Ref.	Ref.	Ref.	Ref.
	N1	1.295(0.952-1.761)	0.1	3.658(2.401-5.571)	**<0.001**
	N2	1.751(0.904-3.390)	0.096	5.404(2.368-12.332)	**<0.001**
	N3	7.823(4.040-15.147)	**<0.001**	25.060(11.628-54.010)	**<0.001**
ER	Positive	Ref.	Ref.	Ref.	Ref.
	Negative	0.942(0.777-1.142)	0.546	0.861(0.603-1.228)	0.409
PR	Positive	Ref.	Ref.	Ref.	Ref.
	Negative	0.900(0.752-1.076)	0.246	0.740(0.530-1.032)	0.076

Chemotherapy	yes	Ref.	Ref.	Ref.	Ref.
	no	1.206(0.930-1.564)	0.158	3.801(2.663-5.425)	**<0.001**
Surgical method	BCS+RT	0.731(0.612-0.875)	**0.001**	0.454(0.319-0.647)	**<0.001**
	Mastectomy	Ref.	Ref.	Ref.	Ref.

OS= overall survival; BCSS = breast cancer-specific survival

Supplementary Table 2 Prognostic factors for OS and BCSS in univariate analysis after PSM

Characteristics		OS		BCSS	
		HR(95%CI)	*P*	HR(95%CI)	*P*
Year of diagnosis	2000-2007	Ref.	Ref.	Ref.	Ref.
	2008-2014	0.809(0.598-1.094)	0.169	0.905(0.521-1.570)	0.722
Age (years)	20-49	Ref.	Ref.	Ref.	Ref.
	50-80	3.732(2.547-5.467)	**<0.001**	0.984(0.596-1.624)	0.948
Race	White	Ref.	Ref.	Ref.	Ref.
	Black	1.744(1.290-2.358)	**<0.001**	2.472(1.436-4.255)	**0.001**
	Other	0.468(0.273-0.802)	**0.006**	0.434(0.136-1.386)	0.159
Marital status	Married	Ref.	Ref.	Ref.	Ref.
	Not married	0.533(0.428-0.665)	**<0.001**	0.724(0.458-1.144)	0.167
Grade	I	Ref.	Ref.	Ref.	Ref.
	II	0.863(0.643-1.157)	0.325	1.599(0.754-3.388)	0.221
	III	0.845(0.627-1.137)	0.266	2.190(1.055-4.548)	**0.035**
	IV	0.745(0.433-1.283)	0.289	1.558(0.479-5.065)	0.461
Nodal status	N0	Ref.	Ref.	Ref.	Ref.
	N1	1.174(0.673-2.046)	0.573	5.078(2.670-9.658)	**<0.001**
	N2	2.772(0.889-8.643)	0.079	13.189(4.139-42.030)	**<0.001**
	N3	7.129(0.999-50.848)	**0.05**	35.904(4.954-260.244)	**<0.001**
ER	Positive	Ref.	Ref.	Ref.	Ref.
	Negative	0.905(0.714-1.147)	0.409	0.903(0.558-1.464)	0.68

PR	Positive	Ref.	Ref.	Ref.	Ref.
	Negative	0.925(0.740-1.155)	0.49	0.773(0.494-1.210)	0.261
Chemotherapy	yes	Ref.	Ref.	Ref.	Ref.
	no	1.046(0.671-1.628)	0.844	3.686(2.094-6.486)	**<0.001**
Surgical method	BCS+RT	0.678(0.542-0.848)	**0.001**	0.568(0.357-0.904)	**0.017**
	Mastectomy	Ref.	Ref.	Ref.	Ref.

OS= overall survival; BCSS = breast cancer-specific survival
